# Functional and Topological Properties in Hepatocellular Carcinoma Transcriptome

**DOI:** 10.1371/journal.pone.0035510

**Published:** 2012-04-23

**Authors:** Ignat Drozdov, Jan Bornschein, Thomas Wex, Najl V. Valeyev, Sophia Tsoka, Peter Malfertheiner

**Affiliations:** 1 British Heart Foundation Centre of Excellence, King's College London, London, United Kingdom; 2 Centre for Bioinformatics, Department of Informatics, School of Natural and Mathematical Sciences, King's College London, London, United Kingdom; 3 Department of Gastroenterology, Hepatology and Infectious Diseases, Otto-von-Guericke-University of Magdeburg, Magdeburg, Germany; 4 Centre for Systems, Dynamics and Control, College of Engineering, Mathematics and Physical Sciences, University of Exeter, Exeter, United Kingdom; Broad Institute of Massachusetts Institute of Technology and Harvard University, United States of America

## Abstract

Hepatocellular carcinoma (HCC) is a leading cause of global cancer mortality. However, little is known about the precise molecular mechanisms involved in tumor formation and pathogenesis. The primary goal of this study was to elucidate genome-wide molecular networks involved in development of HCC with multiple etiologies by exploring high quality microarray data. We undertook a comparative network analysis across 264 human microarray profiles monitoring transcript changes in healthy liver, liver cirrhosis, and HCC with viral and alcoholic etiologies. Gene co-expression profiling was used to derive a consensus gene relevance network of HCC progression that consisted of 798 genes and 2,012 links. The HCC interactome was further confirmed to be phenotype-specific and non-random. Additionally, we confirmed that co-expressed genes are more likely to share biological function, but not sub-cellular localization. Analysis of individual HCC genes revealed that they are topologically central in a human protein-protein interaction network. We used quantitative RT-PCR in a cohort of normal liver tissue (*n* = 8), hepatitis C virus (HCV)-induced chronic liver disease (*n* = 9), and HCC (*n* = 7) to validate co-expressions of several well-connected genes, namely ASPM, CDKN3, NEK2, RACGAP1, and TOP2A. We show that HCC is a heterogeneous disorder, underpinned by complex cross talk between immune response, cell cycle, and mRNA translation pathways. Our work provides a systems-wide resource for deeper understanding of molecular mechanisms in HCC progression and may be used further to define novel targets for efficient treatment or diagnosis of this disease.

## Introduction

Hepatocellular carcinoma (HCC) is the third most common cause of cancer-related death worldwide [1]. Annually, more than 620000 new patients are diagnosed with this disease and 1-year survival rates remain less than 50% [2]. In 70–90% of cases, HCC develops on a background of chronic liver disease, such as chronic inflammation or cirrhosis [3]. Worldwide, the main risk factor for HCC is viral hepatitis, which accounts for 75% of all cases and results in a 20-fold HCC risk increase in affected patients with cirrhosis [4]. In the Western world, additional factors such as alcohol abuse or metabolic disorders (e.g. long-lasting diabetes mellitus) contribute to increasing incidence rates [5], [6]. A precise molecular understanding of pathological transformations in the liver remains the key to successful management of this disease.

The advent of high-throughput technologies (e.g. transcript/protein chips, semi-automated yeast two-hybrid screens) has allowed simultaneous interrogation of multiple molecular components at any given time. Advances in gene expression profiling have been used to identify key differentially expressed genes and their pathways in HCC. For example, a high-density cDNA array was used to correlate overexpression of vimentin (VIM) with metastatic spread [7]. Additionally, integration of chromosome aberrations with microarray and proteomic studies has also led to the development of the first oncogenic HCC database [8]. While these studies have improved our molecular understanding of HCC, it is becoming increasingly clear that a single function can only rarely be attributed to a particular gene or protein [9], [10]. Thus, a deeper understanding of multifactorial mechanisms in HCC progression may be achieved using a systems-wide approach. This shift in paradigm is attracting substantial interest and has been applied successfully to define signaling cascades in cardiac hypertrophy [11], mechanisms of gastric cancer progression [12], and fundamental organizational properties of metabolic networks [13]. More recently, integration of HCC gene expression with topological features of human protein interaction networks resulted in enhanced diagnosis of HCC [14], while network analysis of hepatitis C virus (HCV)-induced HCC elucidated dysfunctional interactions among proteins and aberrant relationships between transcription factors and their target genes [15].

In this study, we expand on the current network-based interpretation of HCC pathogenesis and undertake a computational approach to explore functional and topological properties of key genes and pathways in HCC gene relevance networks derived from 264 human microarray experiments monitoring normal liver tissue, liver cirrhosis, and HCC. Our gene co-expression analysis resulted in a consensus disease network that consisted of 798 genes and 2012 links. Network clustering approach based on gene co-expression patterns identified gene clusters involved in immune response, cell cycle, and mRNA translation pathways. Subsequent analysis using RT-PCR validated co-expressions of several well-connected genes localized to the cell cycle cluster, ASPM (asp [abnormal spindle] homolog, microcephaly associated [Drosophila]), CDKN3 (cyclin-dependent kinase inhibitor 3), NEK2 (NIMA [never in mitosis gene a]-related kinase 2), RACGAP1 (Rac GTPase activating protein 1), and TOP2A (topoisomerase [DNA] II alpha 170 kDa). Taken together, our results present a resource for further identification and validation of potential target genes implicated in HCC pathogenesis.

## Results

### Inference of gene co-expression networks

We investigated gene expression profiles of normal liver tissue, hepatitis B/C- (HBV, HCV) and alcohol-induced cirrhosis, and HCC tumors at various stages of disease and heterogeneous backgrounds (HCV/HBV, alcohol) (**Table 1**). To establish phenotype specificity, we also studied gene expression in breast, colon, and prostate cancers, as well as normal human tissue, obtained from the Genome Novartis Foundation's SymAtlas [16], which contains 79 different normal human tissues (such as liver, brain, and heart) and cell types with 2 replicates per tissue/cell type.

**Table 1:** Summary of microarray datasets.

Microarray Dataset Name	Experimental Conditions	Platform	Number of Probes	Number of Probes after preprocessing	ArrayExpress Accession Number
HCC Dataset [82]	Normal Liver, HBV/HCV/Alcoholic Cirrhosis, HCC N = 65	HG-U133A	22283	13149	E-TABM-36
HCV Dataset [66]	Normal Liver, HCV-HCC N = 124	HG-U133A_2	22277	13149	E-GEOD-14323
Progression Dataset [44]	Normal Liver, HCV-Cirrhosis, 4 stages of HCV-HCC N = 75	HG-U133A Plus 2	54675	21358	E-GEOD-6764
Breast Dataset [68]	Normal, breast tumor N = 86	HG-U133A	22283	13149	E-GEOD-15852
Colon Dataset [69]	Normal, colon cancer N = 47	HG-U133A	22283	13149	E-MTAB-57
Prostate Dataset [70]	Normal, prostate cancer N = 154	HG-U133 Plus 2	54675	21359	E-GEOD-17951
Normal Dataset [16]	Normal tissue and cell lines N = 158	HG-U133A	22283	13149	E-TABM-145

HCC = Hepatocellular Carcinoma; HBV = Hepatitis B Virus, HCV = Hepatitis C Virus.

These additional microarray datasets were selected for the following reasons: 1) breast, colon, and prostate cancers are among the most common global cancer-related malignancies [17]; 2) due to the viral or alcoholic etiology and liver-specificity of HCC tumors, non-liver cancers may be suitable to confirm phenotype-specificity; 3) all microarray datasets represent highly comprehensive normal and cancerous tissue gene expression profiles that are available to the scientific community.

For each dataset, genes whose expression correlated above a predefined Pearson correlation coefficient (PCC) threshold were represented as nodes in an undirected and unweighted network, while their co-expressions formed network edges. To select an appropriate PCC threshold, we compared clustering coefficients of real and random networks for 0.50≤PCC≤1.0 [18] (see **Methods, Suppl. Figure S1**). Gene co-expression networks were constructed from HCC datasets (HCC-Net, HCV-Net, Progression-Net), cancer datasets (Breast-Net, Colon-Net, Prostate-Net), and normal human tissue (Normal-Net) (see **Methods**, **Table 2**). Although, a high confidence normal tissue gene co-expression network already exists [19], we did not use it for comparison to tumor networks to preserve consistency in network reconstruction for all datasets. On average, all networks had large clustering coefficients than what was expected by chance (**Suppl. Figure S1**, mean = 0.45, standard deviation = 0.06), network diameters (mean = 15, standard deviation = 2.9), and assortativities (mean = 0.24, standard deviation = 0.05) (**Table 2**).

**Table 2:** Measures of graph structure and topology across various tumor and normal networks.

Network	PCC cutoff	Nodes	Edges	Average degree	Clustering coefficient	Diameter	Fraction of nodes in giant component	Assortativity
**HCC-Net**	0.76	3155	20975	13.1	0.38	19	0.78	0.31
**HCV-Net**	0.73	6661	134735	40.5	0.44	16	0.96	0.15
**Progression-Net**	0.65	13801	549499	79.6	0.40	14	0.98	0.23
**Breast-Net**	0.66	8918	351819	78.9	0.44	12	0.99	0.25
**Colon-Net**	0.75	7889	265520	67.3	0.41	15	0.98	0.20
**Prostate-Net**	0.59	16955	2703937	318.9	0.54	11	0.99	0.23
**Normal-Net**	0.77	5483	114126	41.6	0.52	18	0.93	0.28
**Conserved-Net**	NA	798	2012	5.0	0.32	10	0.28	0.49

HCC = Hepatocellular Carcinoma; HBV = Hepatitis B Virus, HCV = Hepatitis C Virus, PCC = Pearson correlation coefficient.

**Figure 1:**
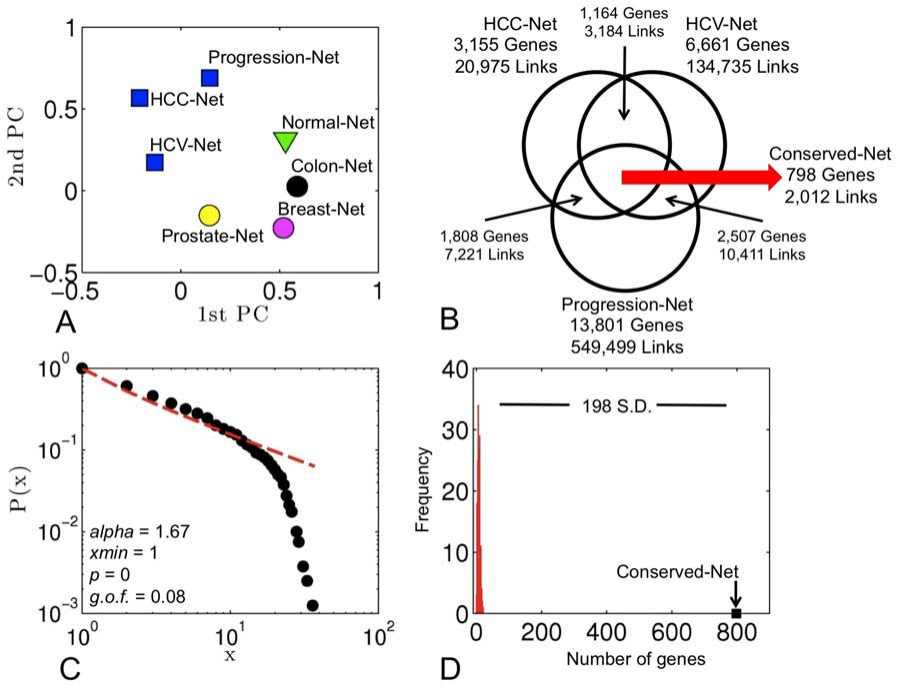
Properties of gene co-expression networks. **A**) Principal component analysis (PCA) of connectivity patterns in HCC datasets and breast, colon, and prostate cancers as well as the normal human tissue. **B**) Venn diagram of conserved genes and links in three HCC datasets with different etiologies. **C**) The cumulative distribution function *P(x)* and the maximum likelihood power-law fit for the node degree distribution in the Conserved-Net. The *alpha* parameter represents the scaling exponent in the power-law fit, *p(x)∼x^−alpha^*, while *xmin* represents the lower cut-off for the scaling region. The corresponding *p* and goodness-of-fit (g.o.f.) values were computed according to the method described in [22]. The red line represents visualization of a power-law distribution of the form *p(x)∼x^−alpha^* for *x≥xmin*. **D**) Comparison of Conserved-Net to intersections of randomly generated networks.

Since it is likely that inter- and intra-cellular connectivity patterns of genes and their products may yield distinct phenotypes [20], [21], we investigated whether phenotypes associated with each gene co-expression network may be distinguished through their respective node degree distributions. To test this hypothesis, node degrees of 762 genes that were common to all gene co-expression networks (HCC-Net, HCV-Net, Progression-Net, Breast-Net, Colon-Net, Prostate-Net, Normal-Net) were projected onto two principal components (PCs). This analysis demonstrated that the HCC interactomes clustered together and were clearly separated from other cancers as well as the normal human interactome, in terms of their node connectivity properties, suggesting phenotype-specificity captured by distinct co-expression patterns (**Figure 1A**).

### Conserved co-expression network in hepatocellular carcinoma

It was previously established that the conservation of gene co-expressions across multiple microarray experiments would improve reliability of network inference [12]. Thus, recurrence of a co-expression link in all three HCC networks was evaluated. The HCC-Net and HCV-Net shared 1164 genes and 3184 links, the HCC-Net and Progression-Net shared 1808 genes and 7221 links, and the HCV-Net and Progression-Net shared 2507 genes and 10411 links (**Figure 1B**). There were 798 genes and 2012 links that were common to all three networks – termed the “Conserved-Net” hereafter (**Figure 1B** **, Suppl. Table S1**, **Suppl. Table S2**).

To test if the node degree distribution of the Conserved-Net follows a power-law, *p(x)*∼*x^−alpha^*, we applied maximum-likelihood fitting methods with goodness-of-fit tests based on the Kolmogorov-Smirnov statistic and likelihood ratios, described previously [22]. The *alpha* value of the power-law fit was estimated to be 1.67, while the lower cut-off, *xmin*, for the scaling region was 1. The *p*-value for the fit to the power-law model between node degree distributions and the power-law was calculated using the approach described in [22]. If the resulting *p*-value is greater than 0.1, the power-law is a plausible hypothesis for the data, otherwise it is rejected. For the Conserved-Net, the *p*-value for the power-law fit was 0. Thus, there is a no support for the power-law node degree distribution in the Conserved-Net (**Figure 1C**).

To ensure that the Conserved-Net intersections are statistically significant, we randomized the HCV-Net, HCC-Net, and Progression-Net by shuffling the edges of each network 538940, 83900, and 2197996 times respectively, while preserving the original node degrees of all nodes (Maslov-Sneppen [MS] model [23]). This procedure was repeated 200 times (see **Methods**), while calculating the number of common genes and links between the randomized networks for all iterations. On average, intersections of randomized HCC networks contained 7.9 genes (standard deviation = 3.9) and 4.1 links (standard deviation = 2.1). Thus, identification of 798 genes and 2012 links in the Conserved-Net was significantly non-random (z-score = 198, **Figure 1D**).

Next, the coverage of Conserved-Net, in terms of the number of oncogenes present, was estimated by searching for genes with known somatic mutations implicated in carcinogenesis. We used the Catalogue of Somatic Mutations in Cancer (COSMIC) [24] as reference and determined that of the 798 genes in the Conserved-Net, 410 (51%) had documented roles in tumorigenesis.

### Topological properties of the Conserved-Net

**Figure 2:**
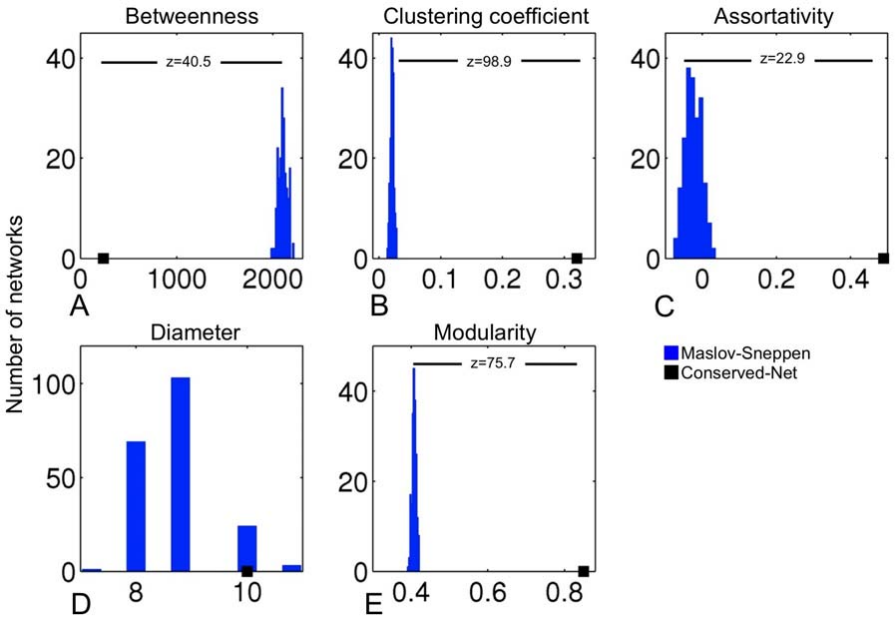
Topological properties of the Conserved-Net. Average betweennes, clustering coefficient, as well as assortativity, diameter, and modularity of the Conserved-Net compared to 200 Markov-Sneppen (MS) random graphs (**A–E**). Real node degrees were not significantly different from random networks. Betweenness centrality, clustering coefficients, assortativity, and modularity of the Conserved-Net were significantly different to those observed in random models (z-scores = 40.5, 98.9, 22.9, 75.5, respectively).

To evaluate topological organization of the Conserved-Net further, we assessed network betweenness, clustering coefficients, assortativity, diameter, and modularity. These parameters were also measured in 200 random networks obtained using the MS model (see **Methods**). Real node degrees and network diameters were not significantly different from random networks. This was expected since the MS algorithm preserves original node degree distribution of the original network. Nevertheless, betweenness, clustering coefficients, assortativity, and modularity of the Conserved-Net were significantly different to those observed in random models (**Figure 2 A–E**).

Interestingly, the Conserved-Net was characterized by a substantially smaller average node degree compared to individual HCC networks (Conserved-Net [average degree = 5.0] vs. HCC-Net [average degree = 13.1], HCV-Net [average degree = 40.5], Progression-Net [average degree = 79.6], **Table 2**), which may indicate a greater degree of fragmentation in the consensus network. Network fragmentation was previously assessed by calculating the fraction of nodes that belong to the largest connected component of a network [25], [26]. Smaller connected components indicate more fragmented networks. The fraction of nodes that formed the largest connected component of the Conserved-Net was 0.28, compared to 0.78, 0.96, and 0.98 for HCV-Net, HCC-Net, and Progression-Net respectively (**Table 2**), supporting the notion that the Conserved-Net is fragmented.

**Figure 3:**
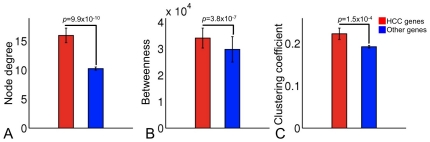
Topological properties of 798 genes of the Conserved-Net in the high-confidence human protein-protein interaction (PPI) network. **A–C**) Average node degree, betweenness centrality, and clustering coefficient of HCC-associated genes derived from the Conserved-Net compared to other genes in the human PPIN. Error bars indicate standard error of the mean (SEM). Statistical significance was calculated using Wilcoxon rank sum test.

Given that biologically essential genes may encode proteins that localize centrally in a protein-protein interaction network (PPIN) [27], we mapped 798 Conserved-Net genes (HCC genes) onto a high-confidence human PPIN (see **Methods**) consisting of 57228 interactions among 11203 proteins. We then calculated node degrees, betweenness centralities, and clustering coefficients of 468 (59%) genes that could be mapped into the PPIN. All topological values of mapped HCC genes were significantly larger than remaining genes in the PPIN (**Figure 3A–C**), while cyclin-dependent kinase 1 (CDKN1) was the only hub node in both the Conserved-Net (node degree = 26) and network of PPIs (node degree = 86). Of note, while 468 HCC genes could be mapped to the PPIN, 216 (46%) of these contained 446 direct protein-protein interactions and could be enriched for GO-Fat terms ‘Translation’, ‘Wound healing’, ‘Immune response’, ‘Cell cycle’, and ‘Negative regulation of apoptosis’.

### Biological interpretation of the Conserved-Net

To provide insight into molecular pathways that are reflected by the 798 genes in the Conserved-Net, we used the DAVID functional enrichment tool to identify over-represented Biocarta, KEGG, and Reactome pathways (see **Methods**). The most abundant pathways were Reactome pathways that include ‘Cell Cycle, Mitotic’ (*n* = 37 genes, *p* = 1.38×10^−4^), ‘Metabolism of proteins’ (*n* = 36 genes, *p* = 1.36×10^−7^), and ‘Signaling in Immune system’ (*n* = 30 genes, *p* = 0.0072) (**Table 3**). Other pathways largely represented genes involved in immune response, translation, and remodeling of the extracellular matrix.

**Table 3:** Functional enrichment of the 798 genes in the Conserved-Net for Reactome, KEGG, and Biocarta pathways.

Category	Term	Count	*P*-value	Benjamini
Reactome Pathway	REACT_152:Cell Cycle, Mitotic	37	1.38×10^−4^	0.0027
Reactome Pathway	REACT_17015:Metabolism of proteins	36	1.36×10^−7^	8.04×10^−6^
Reactome Pathway	REACT_6900:Signaling in Immune system	30	0.0072	0.082
Reactome Pathway	REACT_1762:3′ -UTR-mediated translational regulation	23	6.55×10^−7^	1.93×10^−5^
Reactome Pathway	REACT_6167:Influenza Infection	23	3.10×10^−4^	0.0046
KEGG Pathway	hsa04060:Cytokine-cytokine receptor interaction	22	0.020	0.33
KEGG Pathway	hsa03010:Ribosome	21	4.86×10^−9^	6.91E^−7^
KEGG Pathway	hsa04062:Chemokine signaling pathway	19	0.0051	0.11
KEGG Pathway	hsa04510:Focal adhesion	18	0.022	0.32
KEGG Pathway	hsa05322:Systemic lupus erythematosus	17	2.40×10^−5^	0.0011
KEGG Pathway	hsa04110:Cell cycle	17	4.12×10^−4^	0.012
KEGG Pathway	hsa04610:Complement and coagulation cascades	16	9.46×10^−7^	6.72×10^−5^
KEGG Pathway	hsa04512:ECM-receptor interaction	15	5.51×10^−5^	0.0020
Reactome Pathway	REACT_1538:Cell Cycle Checkpoints	15	0.011	0.10
Reactome Pathway	REACT_13552:Integrin cell surface interactions	11	0.032	0.21
Reactome Pathway	REACT_16888:Signaling by PDGF	10	0.019	0.15
KEGG Pathway	hsa04540:Gap junction	10	0.032	0.34
KEGG Pathway	hsa05130:Pathogenic Escherichia coli infection	8	0.022	0.29
KEGG Pathway	hsa04672:Intestinal immune network for IgA production	7	0.0332	0.33
BIOCARTA	h_tcytotoxicPathway:T Cytotoxic Cell Surface Molecules	6	4.10×10^−4^	0.057
KEGG Pathway	hsa05020:Prion diseases	6	0.028	0.33
BIOCARTA	h_extrinsicPathway:Extrinsic Prothrombin Activation Pathway	5	0.0030	0.19
BIOCARTA	h_thelperPathway:T Helper Cell Surface Molecules	5	0.0043	0.18
BIOCARTA	h_amiPathway:Acute Myocardial Infarction	5	0.0059	0.19
BIOCARTA	h_stathminPathway:Stathmin and breast cancer resistance to antimicrotubule agents	5	0.0059	0.19
BIOCARTA	h_Ccr5Pathway:Pertussis toxin-insensitive CCR5 Signaling in Macrophage	5	0.0079	0.21
BIOCARTA	h_LairPathway:Cells and Molecules involved in local acute inflammatory response	5	0.013	0.27
BIOCARTA	h_fibrinolysisPathway:Fibrinolysis Pathway	4	0.025	0.40
BIOCARTA	h_classicPathway:Classical Complement Pathway	4	0.032	0.44

Terms are sorted by the number of representative genes (Count). Enrichment *p*-values (*P*-value) which were then adjusted using Benjamini-Hochberg multiple testing correction.

An important characteristic of most biological networks is that they tend to naturally organize into modules [28]. We tested whether genes that localized to the same module in the Conserved-Net share biological function. We calculated the number of common functional terms (GO-BP, GO-CC, Reactome pathways) between co-expressed genes in clusters identified by either MCL or Louvain clustering algorithms and compared them to randomly generated clusters with identical number of genes and links. Two independent clustering algorithms were chosen to ensure validity of our observations and functional similarity between all genes was expressed using the Jaccard coefficient (see **Methods**).

**Figure 4:**
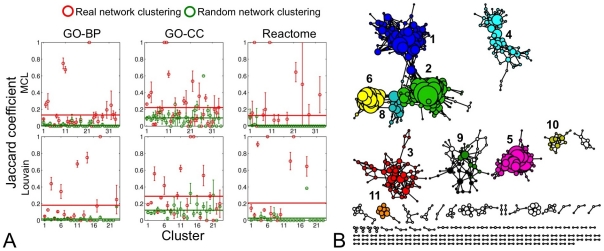
Functional enrichment of the Conserved-Net. **A**) Jaccard functional similarity coefficient between co-expressed greater in MCL and Louvain partitions compared to random partitions with identical numbers of nodes and edges. **B**) Visualization of the Conserved-Net where each node corresponds to a gene and links correspond to conserved co-expressions. Node colors illustrate cluster assignments produced by the Louvain algorithm and node sizes are proportional to node degree.

The MCL algorithm partitioned the Conserved-Net into 247 clusters (modularity = 0.53). The largest cluster contained 30 genes, while the smallest contained 2 genes. Conversely, the Louvain algorithm partitioned the Conserved-Net into 139 clusters (modularity = 0.85) with 89 and 2 genes in the largest and smallest clusters respectively. Both clustering algorithms produced high functional similarity values for GO-BP (12.3 times greater Jaccard coefficient [MCL vs. Random], 10.9 times greater Jaccard coefficient [Louvain vs. Random]) and Reactome (18.2 times greater Jaccard coefficient [MCL vs. Random], 7.7 times greater Jaccard coefficient [Louvain vs. Random]) terms (**Figure 4A**) compared to respective random partitions. Interestingly, GO-CC similarities were not substantially higher in real modules compared to random (2.3 times greater Jaccard coefficient [MCL vs. Random], 2.4 times greater Jaccard Coefficient [Louvain vs. Random]).

Next, due to high functional similarities within partitions, parameter-independent clustering results, and high modularity of identified clusters we selected results produced by the Louvain algorithm for further functional exploration of the Conserved-Net (**Figure 4B**). Enrichment for over-represented GO-BP terms in gene clusters with >10 genes, revealed presence of processes involving ‘Cell cycle’ (*p* = 6.80×10^−29^, Cluster 5), ‘Translation elongation’ (*p* = 2.10×10^−16^, Cluster 3), and ‘Extracellular matrix organization’ (*p* = 1.23×10^−9^, Cluster 1) processes (**Table 4**).

**Table 4:** Functional enrichment of clusters with at least 10 genes for over-represented Gene Ontology (GO)-Biological Process (BP) terms.

GO-BP terms	Cluster	*P*-value
Extracellular matrix organization	1	1.23×10^−9^
Antigen processing and presentation of peptide or polysaccharide antigen via MHC class II	2	1.85×10^−8^
Translational elongation	3	2.10×10^−16^
Regulation of transcription, DNA-dependent	4	1.68×10^−3^
Cell cycle	5	6.80×10^−29^
Immune response	6	2.45×10^−3^
Sensory perception of smell	7	2.21×10^−3^
Cell surface receptor linked signal transduction	8	3.00×10^−5^
Metabolic process	9	2.95×10^−3^
Transcription initiation from RNA polymerase II promoter	10	8.70×10^−4^
Antigen processing and presentation of peptide antigen via MHC class I	11	1.53×10^−9^

### Contribution of the normal liver to the Conserved-Net

It was of interest to investigate to what extent gene co-expression modules in the Conserved-Net were contributed by the normal liver tissue. Results may reveal conserved gene clusters that could represent important oncogenic pathways.

**Figure 5:**
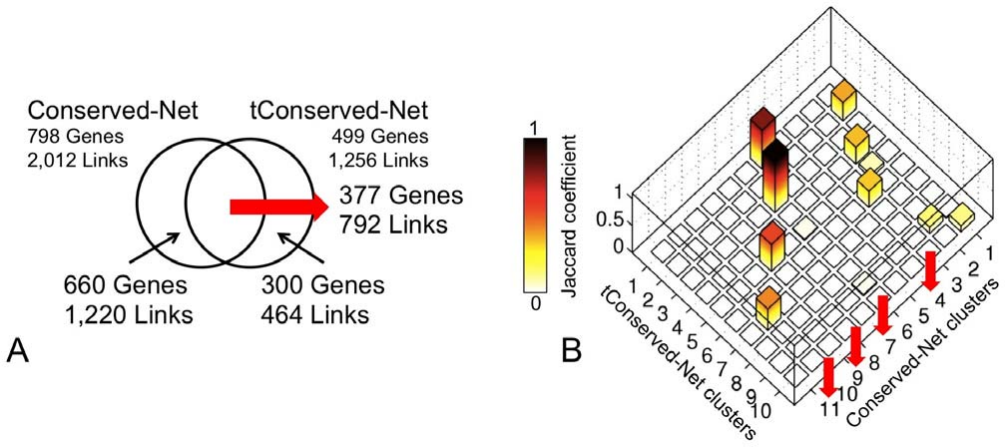
Comparison of Conserved-Net with consensus co-expression network generated by exclusion on normal liver tissue from HCC datasets (tConserved-Net). **A**) Venn diagram visualizing intersection and differences of Conserved-Net and t-Conserved-Net. **B**) Bar graph of Jaccard coefficients that reflect the overlap between gene sets assigned to either Conserved-Net or tConserved-Net clusters by the Louvain algorithm. Only gene clusters with more than 10 genes were used in the analysis. Higher Jaccard coefficient value is indicative of a greater conservation of a cluster between Conserved-Net and tConserved-Net. Red arrows indicate gene clusters that were lost in the absence of normal liver tissue.

To identify conserved gene modules, we repeated gene co-expression analysis of the HCC datasets in the absence of the normal liver profiles. Co-expression networks were constructed by selecting the appropriate PCC cutoff (**Suppl. Figure S2**), as described in the previous sections, and retaining genes and co-expressions conserved in all three HCC microarray datasets. The resulting consensus network of gene co-expressions in malignant samples (tConserved-Net) contained 499 genes and 1256 links. Of these, 377 genes (47%) and 792 links (40%) were shared with the Conserved-Net, while 300 genes and 464 links were specific only to the tConserved-Net (**Figure 5A**). Overall, 407/499 (82%) genes of tConserved-Net were also present in the Conserved-Net, while 92/499 (18%) were specific to the tConserved-Net.

The Louvain algorithm partitioned the tConserved-Net into 96 clusters (modularity = 0.79), with the largest cluster containing 66 genes. Subsequently, we used the Jaccard coefficient to measure the overlap between gene sets assigned to either Conserved-Net or tConserved-Net clusters (**Figure 5B**). Among the cluster with more than 10 genes, clusters 5 (‘Cell cycle’, Jaccard coefficient = 0.72), 6 (‘Immune response’, Jaccard coefficient = 0.93), and 8 (‘Cell surface receptor linked signal transduction’, Jaccard coefficient = 0.50) of the Conserved-Net were preserved the most in the tConserved-Net. Conversely, clusters 4 (‘Regulation of transcription, DNA-dependent’), 7 (‘Sensory perception of smell’), 9 (‘Metabolic process’), and 11 (‘Antigen processing and presentation of peptide antigen via MHC class I’) were eliminated in the tConserved-Net.

### RT-PCR validation

Following the computational analyses of HCC-relevant gene network topologies, we used quantitative RT-PCR in a cohort of normal liver tissue (elevated transaminases but without underlying liver disease or structural changes of the tissue, *n* = 8), HCV-induced chronic liver disease (*n* = 9), and HCC (*n* = 7) to identify transcripts and validate co-expressions of five well-connected genes, namely ASPM (node degree = 6), CDKN3 (node degree = 21), NEK2 (node degree = 14), RACGAP1 (node degree = 11), and TOP2A (node degree = 31) in the ‘Cell cycle’ cluster of Conserved-Net.

**Figure 6:**
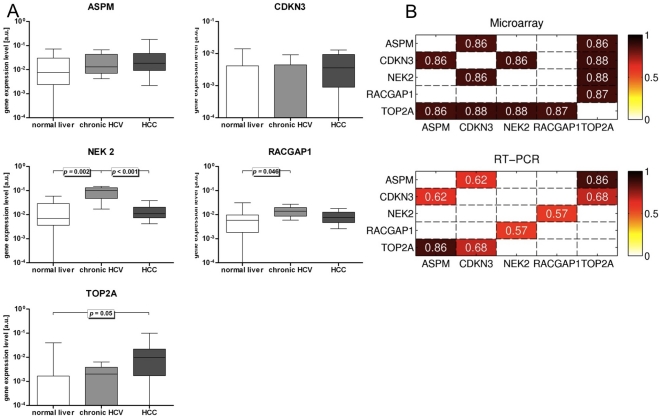
RT-PCR validation of co-expression links among topologically central genes in the ‘Cell cycle’ cluster of the Conserved-Net. **A**) Expression of ASPM, CDKN3, NEK2, RACGAP1, and TOP2A. Illustrated P-values are based on pairwise comparisons by Mann-Whitney U tests, if global Kruskal-Wallis test revealed significant differences (*p*<0.05). **B**) Heatmaps visualizing comparison of average gene co-expressions in HCC microarray datasets with respective transcript co-expressions assessed using RT-PCR. Numbers on each heatmap represent Pearson Correlation Coefficients (PCC). Only statistically significant PCCs were reported for RT-PCR transcripts. *P*<0.05 was considered significant.

At the transcript expression level, statistical significance was noted for RACGAP1, NEK2, and TOP2A (**Figure 6A**). RACGAP1 transcript levels were elevated only slightly (*p* = 0.059) in HCC tissue compared with normal liver, whereas the difference was significant (*p* = 0.046) in fibrotic tissue compared to normal liver. NEK2 expression levels were significantly elevated in fibrotic tissue of Hepatitis C patients compared to both normal controls (*p* = 0.002) and HCC (*p*<0.001). Finally, TOP2A was significantly upregulated in HCC compared to normal liver (*p* = 0.05).

At the transcript co-expression level, statistically significant confirmation of links in the Conserved-Net was achieved for all genes with the exception of NEK2 and RACGAP1 (**Figure 6B, 6C**). The highest PCCs were observed between ASPM and TOP2A (PCC = 0.86, *p* = 1.4×10^−8^) and CDKN3 and TOP2A (PCC = 0.68, *p*<0.001). Unexpected correlation was noted between NEK2 and RACGAP1 (PCC = 0.57, *p* = 0.004). Overall, although some transcript expression differences between normal and HCV/HCC tissues were not statistically significant, presence of 3/6 co-expressions and absence of 3/3 co-expressions were validated by RT-PCR. As such our study offers a preliminary resource for potential gene regulatory patterns that may be validated further in larger patient cohorts.

## Discussion

This study provides an overview of the structure and function of gene co-expression networks across relevant microarray experiments monitoring gene expression in normal liver, liver cirrhosis, and HCC. The primary goal of this work was to describe network topology and identify genome-wide biological mechanisms associated with pathogenesis of HCC. Importantly, results of our work offer a phenotype-specific genomic resource that may be used for further validation in a laboratory setting.

Since threshold selection is a critical aspect in network inference studies, we systematically compared topology of real and random networks to identify an appropriate cut off [18]. This approach is particularly useful when multiple microarray experiments were carried out on different platforms. At each PCC threshold, all microarray networks exhibited a high (0.45) clustering coefficient, a typical characteristic of structured graphs, that is also consistent with general behavior of biological networks [29] and topologies detected in protein-protein interaction collections such as STRING [30] and IntAct [31].

Using principal component analysis (PCA), we confirm that connectivity patterns of all HCC networks are phenotype-specific and differ substantially from breast, colon, and prostate cancers as well as the normal human interactome (**Figure 1A**). This projection of network connectivities to uncorrelated PCs was hypothesized to illustrate global topological patterns and differential gene cross-talk which may be indicative of a unique phenotype. Fundamentally, this approach is different to evaluation of gene expression alone, which does not take into account complex relationships between genes within a biological system. Previously, phenotype-specificity was established at the gene expression level [32]. However, such approaches rely on temporal behavior of a single gene across tissues and do not consider systems level relationships between genes. We also note that all cancer networks shared 158 genes and 157 links, suggesting that there may be a general “onco-” signature that may be explored in future studies.

Due to the high-throughput nature of microarray technology and a large number of co-expression links (>700000), it is possible that some of these links are false positives or features of a specific microarray platform. Thus, evaluation of conserved co-expression links across three HCC networks has a number of additional strengths compared to conventional statistical approaches. First, we used an automated method for selection of PCC thresholds, as an objective filtering process to select significant co-expressions that eliminates the necessity to apply a stringent statistical cut off, such as a *p*-value or a fold change [18]. Second, the data was combined at the level of correlation matrices, rather than gene expression levels, which facilitates between-study comparisons [33] and improves functional relevance of co-expressed genes [12]. Finally, reproducible co-expressions are less likely to be false-positives and may reflect a biologically relevant link [34]. For example, in a meta-analysis of >300 tissue samples of gastric cancer, this hypothesis helped to identify a functional link between prognostic marker PLA2G2A and the EphB2 receptor [12].

Due to a heterogeneous etiology of HCC datasets, conserved co-expressions provide an overview of common mechanism in tumors with HCV, HBV, and alcoholic backgrounds. We identify 798 genes and 2012 conserved co-expressions that formed Conserved-Net. This interactome was confirmed to be non-random. Interestingly, of the 798 genes in the Conserved-Net, 331 (41%) had known roles in tumorigenesis. In addition, automated PubMed search for 798 genes in the context of ‘Hepatocellular Carcinoma’, ‘Cancer’, ‘Hepatitis B’, ‘Hepatitis C’, ‘Alcohol’, or ‘Cirrhosis’ identified that 507/798 (64%) genes had at least one matching abstract (data not shown), suggesting that the Conserved-Net provides acceptable coverage of the current molecular knowledge in HCC and may contain some novel tumor-associated genes.

Network topology analysis of the Conserved-Net was undertaken to characterize importance of individual genes within the established interactome. Such systems modeling approaches may be used to define putative drug targets in HCC and improve efficacy of current therapies [35], [36]. First, it was confirmed that the topological properties (betweeneess, clustering coefficients, assortativity, diameter, and modularity) of the Conserved-Net are significantly non-random, thus more likely to reflect biological node importance [37].

The Conserved-Net exhibited a small average node degree compared to individual HCC networks (**Table 2**), likely reflecting fragmentation of the network into disconnected components. Biologically this finding implies that HCC tumors with diverse etiologies (HCV-/HBV-infection, and alcoholic backgrounds) monitored by individual microarray datasets contain surprisingly few shared gene co-expression patterns, suggesting molecular heterogeneity of each etiology [38].

The consensus HCC network was also highly assortative (assortativity = 0.49), suggesting that genes with similar node degrees tend to co-express. Additionally, assortativity of the conserved HCC network was higher compared to individual HCC networks. These results are unexpected, since previous studies report that gene co-expression networks are typically disassortative, i.e. high-degree genes tend to connect to low-degree genes [37], [39]. Recently, increased assortativity was reported to be associated with highly fragmented networks [40], further supporting our observation of fragmentation in the Conserved-Net.

The Conserved-Net was studied at the level of individual genes. For example, CD53 was the most connected gene (36 co-expressions) and has been shown to be associated with metastatic spread in hepatocellular carcinoma [41]. Of interest, ArhGAP15 (Rho GTPase activating protein 15) was noted to be a “bottleneck” gene [27] with the highest betweenness centrality measure in the Conserved-Net (13551). ArhGAP15 is a member of the Rho GTPase activating family of proteins, which act to catalyze the hydrolysis of GTP that is bound to Rho, Rac and/or Cdc42, thus inactivating regulators of the actin cytoskeleton. This family of proteins is emerging as a set of attractive tumor suppressor genes in hepatocellular carcinoma [42], [43]. Of note, there were 6 genes belonging to Rho GTPase family (ArhGAP15, RHOQ, RAC2, ArhGAP25, ECT2, RACGAP1).

Other topologically important genes were determined to be CCNB1 (Cyclin B1, node degree = 41), AEBP1 (adipocyte enhancer binding protein 1, node degree = 26), and TTK (Phosphotyrosine picked threonine-protein kinase, node degree = 20). CCNB1 has been shown to be upregulated in HCV-induced HCC [44], while AEBP1 has been demonstrated to manifest a proinflammatory function by up-regulating NF-kappaB and suggested as a potential therapeutic target for the treatment of various chronic inflammatory diseases and cancer [23]. Finally, TTK is known to be upregulated in anaplastic thyroid carcinoma [18] and associated with increased risk of breast cancer [23]. Of interest, Conserved-Net also identified VIM (vimentin, node degree = 11), a well-characterized marker of epithelial–mesenchymal transition [45], which is also known to be a marker of poor HCC prognosis [46]. Recently, VIM was reported to be upregulated in response to alcohol in Caco-2 (colon cancer), MCF-7 and MDA-MB-231 (breast cancer), and IEC-6 (nontransformed, normal intestinal) cell lines [47] further suggesting the presence of alcohol etiology in the Conserved-Net.

Potential functional importance of 798 genes in the Conserved-NET was explored through mapping them onto a high-confidence human PPIN. Interestingly, for the 468/798 (59%) genes that could be mapped to the PPIN, node degree, betweenness centrality, and clustering coefficients were significantly higher compared to other genes in the human interactome. These findings are consistent with the general behavior of tumor genes [29], [48] and indicate that genes inferred by co-expression analysis may indeed be functionally critical due to their central topologies in the human PPIN [49]. It was also of interest to note that while 468 HCC genes could be traced on the PPIN, 216 (46%) of these contained 446 direct protein-protein interactions and could be enriched for GO-Fat terms ‘Translation’, ‘Wound healing’, ‘Immune response’, ‘Cell cycle’, and ‘Negative regulation of apoptosis’. Our observations indicate that the above processes may be disrupted at the protein-interaction level and are likely to contribute to pathogenesis of HCC.

Delineation of the community structure in interactomes is an important aspect of network biology that may highlight general signaling mechanisms associated with disease [50]. We explored gene cluster architecture of the Conserved-NET using MCL and Louvain clustering algorithms. We confirmed our hypothesis that genes with similar biological function tend to co-localize to the same network module. Interestingly, MCL and Louvain algorithms produced comparable functional homogeneity within respective partitions, suggesting that both methods are useful for identification of functionally relevant network modules. Of note, co-expressed genes tended to have higher GO-BP and Reactome pathways term similarity. This implies that gene co-expression is more likely to occur between functionally related genes rather that between genes with similar subcellular localizations.

Due to high functional similarities within partitions and parameter-independent clustering results, we selected results produced by the Louvain algorithm for further functional exploration of the Conserved-Net. Enrichment of 11 modules (>10 genes each) for GO-BP terms, demonstrated that HCC is a heterogeneous disease that relates to processes such as ‘Cell cycle’, ‘Translation elongation’, and ‘Extracellular matrix organization’. Previously, protein levels and kinase activities of cyclin D1, E, Cdk4, cyclin A, and Wee1 were demonstrated to increase proportionally with the development of HCC, especially in the transition process from chronic hepatitis to HCC [51]. Additionally, 6 genes involved in cell-cycle control and proliferation (BIC, CPNE1, RBPMS, RFC4, RPSA, TOP2A) were among the most significantly upregulated genes both in dedifferentiated HCC and in HCC with loss of chromosomal region 13q [52]. Interestingly, RFC4 (node degree = 1) and TOP2A (node degree = 31) were identified in the Conserved-Net and localized to the ‘Cell cycle’ enriched cluster. These results suggest that gene topological properties may be explored further in a laboratory setting. Previous studies suggest that premalignant transformations in extracellular composition play a key role in pathogenesis of HCC [53]. We support this hypothesis through identifying a cluster of 89 genes and 318 co-expressions enriched for ‘Extracellular matrix organization’. Among these, we identified several proteins associated with extracellular remodeling (MMP2 and ADAMTS1), which were also previously implicated in HCC progression [54], [55]. Finally, identification of genes involved in ‘Translational elongation’, suggests that the mRNA translation program is activated in HCC. Indeed, this is consistent with the long-standing understanding that coordination and specific activation of translation factor genes might be involved in the process of liver carcinogenesis [56]. Thus, gene enrichment reflects the biology of HCC.

To investigate the extent to which gene co-expression clusters in the Conserved-Net were contributed by the normal liver tissue, we have repeated network inference using the HCC microarray datasets in the absence of normal liver tissue. Our findings indicate that 407/499 (82%) genes in the tConserved-Net were also present in the Conserved-Net. These conserved genes represented clusters enriched for ‘Cell cycle’, ‘Immune response’, and ‘Cell surface receptor linked signal transduction’, reiterating the importance of these biological processes in the development of HCC [57]. Surprisingly, only 92/499 (18%) genes were specific to the tConserved-Net, suggesting that only a small fraction of genes is gained by the exclusion of normal liver. As such, 391 genes that are specific to the Conserved-Net were likely contributed by the inclusion of normal liver in the computational analysis.

To experimentally validate co-expressed genes that belong to the ‘Cell cycle’ cluster, we measured transcript levels of 5 highly connected genes in the Cluster 5 - ASPM, CDKN3, NEK2, RACGAP1, and TOP2A using quantitative RT-PCR. Statistically significant co-expressions patterns were confirmed in an independent tissue set between ASPM, CDKN3, and TOP2A. Additionally, we also reported a statistically significant (*p*<0.05) overexpression of TOP2A in HCC tissue compared to normal liver samples. In an analysis of 247 tissue samples from HCC, ASPM was overexpressed in 66% of them, showing an association with vascular invasion, early recurrence and poor prognosis [58]. Similarly, overexpression of TOP2A was associated with advance histological grading, vascular invasion, and an early age onset of HCC [59], while CDKN3 was reported as part of a vascular invasion signature in HCC [60] and has been implicated in other malignancies including breast and prostate cancers [61]. These findings confirm that functionally similar genes tend to co-express across multiple phenotypic conditions. Thus, the Conserved-Net may be further used to characterize biological roles of unknown genes given the functional properties of their co-expressions.

Although we could not validate RACGAP1-TOP2A, NEK2-TOP2A, and CDKN3-NEK2 co-expressions, we noted a statistically significant up-regulation of NEK2 and RACGAP1 in fibrotic/cirrhotic tissue (*p* = 0.002 and *p* = 0.046 respectively). A possible reason for this expression pattern could be an early role of these genes in tumor initiation. NEK2 and RACGAP1 have been shown to be involved in oncogenic pathways and oncogene-regulated processes such as BRCA1- and Wnt-dependent signaling cascades [62], [63], [64], [65], possibly implicating these pathways in the development of HCC.

Overall, the five analyzed genes were expressed in different stages of liver disease, suggesting that consideration of graph topology as well as differential expression may improve detection of putative biomarkers *in vivo*. However, data generated in this study is a preliminary verification of the computational analysis rather than an exhaustive biomarker study, and thus a larger, prospective study is critical.

This report presents a phenotype-specific resource for the study of HCC with diverse etiology and varying degrees of progression. With the rapidly increasing availability of comprehensive “omics” datasets in the public domain, it is likely that analytical approaches described here will be valuable aids to clinicians and researchers aiming at the elucidation of both the general and specific mechanisms of tumor formation. Furthermore, expansion or modification of this methodology may improve sensitivity and specificity of novel diagnostic and prognostic markers and contribute to the identification of appropriate strategies for personalized patient treatment.

## Methods

### Data Preparation

Three publicly available human HCC microarray datasets were included in this study, individually referred to as the HCC Dataset, HCV Dataset, and Progression Dataset (**Table 1**). These datasets corresponded to 264 arrays [44], [66], [67]. In the HCC Dataset, ‘Normal Liver’ refers to 5 pools of non-tumorous liver tissue, specifically alcoholic cirrhosis, alcoholic noncirrhotic liver, HBV noncirrhotic liver, HCV cirrhosis, and HBV cirrhosis. In the HCV Dataset, ‘Normal Liver’ refers to non-tumorous liver tissue obtained from deceased donors. Finally, in the Progression Dataset, ‘Normal Liver’ refers to tissue obtained from the healthy livers of patients undergoing resection for hepatic hemangioma.

Additional datasets, breast [68] (*n* = 86 arrays), colon [69] (*n* = 47 arrays), and prostate [70] (*n* = 154 arrays), as well as normal human tissue arrays [16] (*n* = 158) were obtained to measure phenotype specificity of HCC datasets (**Table 1**). For all datasets raw expression values were downloaded from the ArrayExpress database [71] and normalized using Robust Multi-array Average (RMA) [72]. To standardize annotation across microarray platforms, Affymetrix probe identifiers were mapped to their corresponding Ensembl gene identifiers [73]. Furthermore, to facilitate integration of multiple public datasets, in cases where multiple probesets mapped to a single gene, median intensity values were retained. In this manuscript, networks from datasets TABM-36, E-GEOD-14323, E-GEOD-6764, E-GEOD-15852, E-MTAB-57, E-GEOD-17951, and E-TABM-145 were referred to as HCC-Net, HCV-Net, Progression-Net, Breast-Net, Colon-Net, Prostate-Net, and Normal-Net, respectively.

### Gene network inference

Pairwise similarity in gene expression vectors was expressed by the Pearson correlation coefficient (PCC). Gene pairs that correlated above a predefined PCC threshold were represented in the form of an undirected unweighted network, where nodes correspond to genes and links (edges) correspond to co-expression between genes. To infer the PCC threshold in an unbiased manner, a systematic procedure was used based on the topological difference between real and random co-expression networks [18]. This method is especially useful for integration of multiple microarray studies performed on different platforms.

To confirm non-random behavior of real microarray networks, we compared them to the Maslov-Sneppen (MS) model [23]. All MS networks were generated by rewiring edges in the original networks while preserving the degrees of the respective nodes. The number of rewiring steps taken for each model was 4× (number of edges).

### Graph analysis

Topological properties examined were node degree, betweenness centrality, clustering coefficient, assortativity, diameter, and modularity [74]. Node degree is defined as the total number of edges that connect to a given node. Betweenness centrality is the measure of node importance within a graph. Previously, betweenness centrality was proposed as an indicator of biological significance of a gene [27]. Clustering coefficient is the probability that a node's neighbors are interconnected. Assortativity is the quantity that measures the tendency for nodes with similar magnitude of degrees to be connected by an edge. Network diameter is defined as the largest shortest path between any pair of nodes in the network. Modularity is the strength of community structure in graphs [75]. Network analyses were carried out using the Functional Genomic Assistant (FUGA) toolbox [76]. Statistical significance of differential topology was assessed using the Wilcoxon rank sum test. *P*-values<0.05 were considered significant.

### Network partitioning and assessment of functional similarity

Clusters of genes in a co-expression network were identified by using either the Louvain method, a fast algorithm for community detection in graphs using optimization of modularity [77] or the Markov Cluster Algorithm (MCL), a fast and scalable algorithm based on simulation of stochastic flow in graphs [78].

Functional similarity between co-expressed genes that localized to the same graph partition was measured by the Jaccard similarity coefficient of overlapping Gene Ontology (GO) Biological Process (BP), GO Cellular Component (CC), and Reactome pathways terms. To ensure specificity of annotation within a gene cluster, functional categories containing <5 or >50 genes were removed. For each co-expressed gene pair, the Jaccard coefficient was expressed as the ratio of the number of common functional terms to the number of total functional terms.

### Functional enrichment

For a cluster with *n* genes and an *a priori* defined functional category with *K* genes, the hypergeometric test was used to evaluate the significance of the overlap *k* between the cluster and a Gene Ontology term [79]. All genes in a network were used as reference. Additionally, we used the Database for Annotation, Visualization and Integrated Discovery (DAVID) [80] for statistical enrichment of GO-Fat terms. Given the hierarchical structure of the GO database, GO-Fat terms are manually curated by the DAVID database and attempt to filter the broadest terms so that they do not overshadow the more specific terms.

### Extraction of total RNA and quantitative Real-time Polymerase Chain Reaction (RT-PCR)

The study was approved by the Official Ethics Committee of the Medical Faculty of the Otto-von-Guericke University of Magdeburg and written informed consent to participate in the study was obtained from all subjects included. Liver tissue was obtained by ultrasound-guided fine-needle biopsy from eight patients with HCC (lesional tissue), nine patients with Hepatitis C induced liver fibrosis and from eight patients with elevated transaminases but without underlying liver disease or structural changes of the tissue architecture. Biopsies were snap frozen in liquid nitrogen upon extraction and consequently transferred onto a 1.5 ml RNase-free Eppendorf tube and submerged in 0.5 ml of TRIZOL-reagent and stored at −80°C until processing.

Total RNA was extracted using a “two-step” protocol as described previously [25]. Briefly, a single biopsy was homogenized in 500 µl Trizol using disposable probes with tissue raptor (QIAGEN, Hilden, Germany) on ice. After complete homogenization 200 µl chloroform was added, the sample was extensively vortexed and centrifuged in a microcentrifuge (14000×g, 4°C) for 15 min. The supernatant mixed with equal volume of isopropanol in a new tube, vortexed and incubated on ice for 10 min. Precipitated RNA was obtained by centrifugation (14000×g, 4°C, 10 min), and resolved in 100 µl RNase-free water. Subsequently, the RNA was purified using the RNeasy kit (Qiagen, Hilden, Germany) according to the manufacturer's instruction. Finally, the RNA was eluted in 70 µl RNase-free water. Aliquots of 5 µl each were used for determination of RNA concentration via UV-spectroscopy and to evaluate RNA integrity by agarose gel electrophoresis. In each case, 500 ng of total RNA was transcribed into cDNA in a 40 µl reaction volume by AMV reverse transcriptase (Promega, Mannheim, Germany) and random hexanucleotides (Boehringer, Mannheim, Germany) using standard protocol as described earlier [81].

Quantitative RT-PCR was performed in an iCycler (BioRad, Munich, Germany). The 30 µl reaction mixture contained 10 µl RNase-free water, 15 µl HotStarTaq-Sybr. Green, 0.2 µl of both primers (50 µM) and 1.2 µl c-DNA. Initial denaturation and activation of Taq-polymerase at 95°C for 15 min was followed by 40 cycles. The fluorescence intensity of the double-strand specific SYBR-Green I, reflecting the amount of actually formed PCR-product, was read real-time at the end of each elongation step. Transcript amounts were calculated based on the Ct values of each sample. Arbitrary units reflect the expression of the given gene in relation to β-actin transcript amount. Primers used and the size of expected PCR fragments are listed in **Table 5**.

**Table 5:** Primers used for quantitative RT-PCR analysis.

ASPM-a	AACCCATTATCGCTGTGGCAC (21)
ASPM-b	ACCACCAAGTGAAGCCCTGTTC (22)
CDKN3-a	TCACCCATCATCATCCAATCGC (22)
CDKN3-b	CTCGCAGGCTGTCTATGGCTTG (22)
NEK2-a	CATTGGGCTGCTTGCTGTATG (21)
NEK2-b	TTCTGGCTCTCCTAATTGTCGC (22)
RACGAP1-a	TCTCAACAGAGGCCAACCATCC (22)
RACGAP1-b	ACTGCAGAGCCAATGGAACGAG (22)
TOP2A-a	TTTCAGGCCTTGGTGTGGTTGG (22)
TOP2A-b	TCGCAGAAGAGAGGGCCAGTTG (22)
ß-actin-a	CATGCCATCCTGCGTCTGGACC (22)
ß-actin-b	ACATGGTGGTGCCGCCAGACAG (22)

### Statistical analysis

RT-PCR data was analyzed using SPSS 12.0 (SPSS Inc., Chicago, IL, USA) and graphs were generated using GraphPad Prism 4.0 (GraphPad Software Inc., San Diego, CA, USA). Non-parametric tests were used for statistical analyses of transcript expression values in order to account for possibly skewed distributions. First, the Kruskal-Wallis test was applied to each gene across all groups. In case of a positive test result, the Mann-Whitney U test was performed to carry out pairwise group comparisons. All test were two-tailed with a significance level of *p*<0.05. Pearson correlation coefficient *p*-values for transcript co-expressions were calculated using MATLAB's corrcoef function (2009a, The MathWorks, Natick, MA). Statistical significance of gene network topologies was calculated using Wilcoxon rank sum test and MATLAB implementation of the ranksum function in the Statistics toolbox.

## Supporting Information

Figure S1 **Selection of Pearson correlation coefficient threshold for gene co-expression network inference.** Clustering coefficients of real (black) and random (blue) networks with identical node degree distributions were systematically measured for 0.50≤PCC≤1.0. Threshold was selected at the first local maximum of the difference (red) between the real and random clustering coefficients.
(TIFF)

Figure S2 **Selection of Pearson correlation coefficient threshold for gene co-expression networks of HCC microarrays without normal liver.** Clustering coefficients of real (black) and random (blue) networks with identical node degree distributions were systematically measured for 0.50≤PCC≤1.0. Threshold was selected at the first local maximum of the difference (red) between the real and random clustering coefficients.
(TIFF)

Table S1 **Gene co-expression network in Hepatocellular Carcinoma.**
(XLS)

Table S2 **Topological properties of genes in the consensus Hepatocellular Carcinoma network.**
(XLS)

## References

[1] Parkin DM, Bray F, Ferlay J, Pisani P (2005). Global cancer statistics, 2002.. CA Cancer J Clin.

[2] Altekruse SF, McGlynn KA, Reichman ME (2009). Hepatocellular carcinoma incidence, mortality, and survival trends in the United States from 1975 to 2005.. Journal of clinical oncology: official journal of the American Society of Clinical Oncology.

[3] Schutte K, Bornschein J, Malfertheiner P (2009). Hepatocellular carcinoma–epidemiological trends and risk factors.. Dig Dis.

[4] Moller H, Mellemgaard A, Lindvig K, Olsen JH (1994). Obesity and cancer risk: a Danish record-linkage study.. Eur J Cancer.

[5] Seitz HK, Stickel F (2006). Risk factors and mechanisms of hepatocarcinogenesis with special emphasis on alcohol and oxidative stress.. Biol Chem.

[6] El-Serag HB, Hampel H, Javadi F (2006). The association between diabetes and hepatocellular carcinoma: a systematic review of epidemiologic evidence.. Clin Gastroenterol Hepatol.

[7] Hu L, Lau SH, Tzang CH, Wen JM, Wang W (2004). Association of Vimentin overexpression and hepatocellular carcinoma metastasis.. Oncogene.

[8] Su WH, Chao CC, Yeh SH, Chen DS, Chen PJ (2007). OncoDB.HCC: an integrated oncogenomic database of hepatocellular carcinoma revealed aberrant cancer target genes and loci.. Nucleic Acids Res.

[9] Weng G, Bhalla US, Iyengar R (1999). Complexity in biological signaling systems.. Science.

[10] Hartwell LH, Hopfield JJ, Leibler S, Murray AW (1999). From molecular to modular cell biology.. Nature.

[11] Drozdov I, Tsoka S, Ouzounis CA, Shah AM (2010). Genome-wide expression patterns in physiological cardiac hypertrophy.. BMC Genomics.

[12] Aggarwal A, Guo DL, Hoshida Y, Yuen ST, Chu KM (2006). Topological and functional discovery in a gene coexpression meta-network of gastric cancer.. Cancer Res.

[13] Guimera R, Nunes Amaral LA (2005). Functional cartography of complex metabolic networks.. Nature.

[14] Zhang Y, Wang S, Li D, Zhnag J, Gu D (2011). A systems biology-based classifier for hepatocellular carcinoma diagnosis.. PLoS One.

[15] He B, Zhang H, Shi T (2011). A comprehensive analysis of the dynamic biological networks in HCV induced hepatocarcinogenesis.. PLoS One.

[16] Su AI, Wiltshire T, Batalov S, Lapp H, Ching KA (2004). A gene atlas of the mouse and human protein-encoding transcriptomes.. Proceedings of the National Academy of Sciences of the United States of America.

[17] Jemal A, Bray F, Center MM, Ferlay J, Ward E (2011). Global cancer statistics.. CA: a cancer journal for clinicians.

[18] Elo LL, Jarvenpaa H, Oresic M, Lahesmaa R, Aittokallio T (2007). Systematic construction of gene coexpression networks with applications to human T helper cell differentiation process.. Bioinformatics.

[19] Prieto C, Risueno A, Fontanillo C, De las Rivas J (2008). Human gene coexpression landscape: confident network derived from tissue transcriptomic profiles.. PLoS One.

[20] Schadt EE (2009). Molecular networks as sensors and drivers of common human diseases.. Nature.

[21] Barabasi AL, Gulbahce N, Loscalzo J (2011). Network medicine: a network-based approach to human disease.. Nat Rev Genet.

[22] Clauset A, Shalizi CR, Newman MEJ (2009). Power-law distributions in empirical data.. SIAM Review.

[23] Maslov S, Sneppen K (2002). Specificity and stability in topology of protein networks.. Science.

[24] Forbes SA, Bindal N, Bamford S, Cole C, Kok CY (2011). COSMIC: mining complete cancer genomes in the Catalogue of Somatic Mutations in Cancer.. Nucleic acids research.

[25] Callaway DS, Newman ME, Strogatz SH, Watts DJ (2000). Network robustness and fragility: percolation on random graphs.. Physical review letters.

[26] Buldyrev SV, Parshani R, Paul G, Stanley HE, Havlin S (2010). Catastrophic cascade of failures in interdependent networks.. Nature.

[27] Yu H, Kim PM, Sprecher E, Trifonov V, Gerstein M (2007). The importance of bottlenecks in protein networks: correlation with gene essentiality and expression dynamics.. PLoS Comput Biol.

[28] Rives AW, Galitski T (2003). Modular organization of cellular networks.. Proceedings of the National Academy of Sciences of the United States of America.

[29] Jeong H, Mason SP, Barabasi AL, Oltvai ZN (2001). Lethality and centrality in protein networks.. Nature.

[30] von Mering C, Huynen M, Jaeggi D, Schmidt S, Bork P (2003). STRING: a database of predicted functional associations between proteins.. Nucleic Acids Res.

[31] Hermjakob H, Montecchi-Palazzi L, Lewington C, Mudali S, Kerrien S (2004). IntAct: an open source molecular interaction database.. Nucleic Acids Res.

[32] de Souto MC, Costa IG, de Araujo DS, Ludermir TB, Schliep A (2008). Clustering cancer gene expression data: a comparative study.. BMC Bioinformatics.

[33] Berg J, Lassig M (2006). Cross-species analysis of biological networks by Bayesian alignment.. Proceedings of the National Academy of Sciences of the United States of America.

[34] Lee HK, Hsu AK, Sajdak J, Qin J, Pavlidis P (2004). Coexpression analysis of human genes across many microarray data sets.. Genome Res.

[35] Fliri AF, Loging WT, Volkmann RA (2009). Drug effects viewed from a signal transduction network perspective.. J Med Chem.

[36] Florez AF, Park D, Bhak J, Kim BC, Kuchinsky A (2010). Protein network prediction and topological analysis in Leishmania major as a tool for drug target selection.. BMC Bioinformatics.

[37] Barrenas F, Chavali S, Holme P, Mobini R, Benson M (2009). Network properties of complex human disease genes identified through genome-wide association studies.. PLoS One.

[38] Hoshida Y, Toffanin S, Lachenmayer A, Villanueva A, Minguez B (2010). Molecular classification and novel targets in hepatocellular carcinoma: recent advancements.. Seminars in liver disease.

[39] Xulvi-Brunet R, Li H (2010). Co-expression networks: graph properties and topological comparisons.. Bioinformatics.

[40] Friedel CC, Zimmer R (2007). Influence of degree correlations on network structure and stability in protein-protein interaction networks.. BMC Bioinformatics.

[41] Cheung ST, Chen X, Guan XY, Wong SY, Tai LS (2002). Identify metastasis-associated genes in hepatocellular carcinoma through clonality delineation for multinodular tumor.. Cancer Res.

[42] Durkin ME, Ullmannova V, Guan M, Popescu NC (2007). Deleted in liver cancer 3 (DLC-3), a novel Rho GTPase-activating protein, is downregulated in cancer and inhibits tumor cell growth.. Oncogene.

[43] Wong CM, Yam JW, Ching YP, Yau TO, Leung TH (2005). Rho GTPase-activating protein deleted in liver cancer suppresses cell proliferation and invasion in hepatocellular carcinoma.. Cancer Res.

[44] Wurmbach E, Chen YB, Khitrov G, Zhang W, Roayaie S (2007). Genome-wide molecular profiles of HCV-induced dysplasia and hepatocellular carcinoma.. Hepatology.

[45] Yu G, Li F, Qin Y, Bo X, Wu Y (2010). GOSemSim: an R package for measuring semantic similarity among GO terms and gene products.. Bioinformatics.

[46] Shi Z, Derow CK, Zhang B (2010). Co-expression module analysis reveals biological processes, genomic gain, and regulatory mechanisms associated with breast cancer progression.. BMC Syst Biol.

[47] Forsyth CB, Tang Y, Shaikh M, Zhang L, Keshavarzian A (2010). Alcohol stimulates activation of Snail, epidermal growth factor receptor signaling, and biomarkers of epithelial-mesenchymal transition in colon and breast cancer cells.. Alcohol Clin Exp Res.

[48] Sun J, Zhao Z (2010). A comparative study of cancer proteins in the human protein-protein interaction network.. BMC Genomics.

[49] Zotenko E, Mestre J, O'Leary DP, Przytycka TM (2008). Why do hubs in the yeast protein interaction network tend to be essential: reexamining the connection between the network topology and essentiality.. PLoS Comput Biol.

[50] Stuart JM, Segal E, Koller D, Kim SK (2003). A gene-coexpression network for global discovery of conserved genetic modules.. Science.

[51] Masaki T, Shiratori Y, Rengifo W, Igarashi K, Matsumoto K (2000). Hepatocellular carcinoma cell cycle: study of Long-Evans cinnamon rats.. Hepatology.

[52] Skawran B, Steinemann D, Becker T, Buurman R, Flik J (2008). Loss of 13q is associated with genes involved in cell cycle and proliferation in dedifferentiated hepatocellular carcinoma.. Mod Pathol.

[53] Abdel-Hamid NM (2009). Premalignant variations in extracellular matrix composition in chemically induced hepatocellular carcinoma in rats.. J Membr Biol.

[54] Chen RX, Xia YH, Xue TC, Ye SL (2010). Osteopontin promotes hepatocellular carcinoma invasion by up-regulating MMP-2 and uPA expression.. Mol Biol Rep.

[55] Braconi C, Meng F, Swenson E, Khrapenko L, Huang N (2009). Candidate therapeutic agents for hepatocellular cancer can be identified from phenotype-associated gene expression signatures.. Cancer.

[56] Shuda M, Kondoh N, Tanaka K, Ryo A, Wakatsuki T (2000). Enhanced expression of translation factor mRNAs in hepatocellular carcinoma.. Anticancer Res.

[57] El-Serag HB (2011). Hepatocellular carcinoma.. The New England journal of medicine.

[58] Lin SY, Pan HW, Liu SH, Jeng YM, Hu FC (2008). ASPM is a novel marker for vascular invasion, early recurrence, and poor prognosis of hepatocellular carcinoma.. Clin Cancer Res.

[59] Wong N, Yeo W, Wong WL, Wong NL, Chan KY (2009). TOP2A overexpression in hepatocellular carcinoma correlates with early age onset, shorter patients survival and chemoresistance.. International journal of cancer Journal international du cancer.

[60] Minguez B, Hoshida Y, Villanueva A, Toffanin S, Cabellos L (2011). Gene-expression signature of vascular invasion in hepatocellular carcinoma.. Journal of hepatology.

[61] Lee SW, Reimer CL, Fang L, Iruela-Arispe ML, Aaronson SA (2000). Overexpression of kinase-associated phosphatase (KAP) in breast and prostate cancer and inhibition of the transformed phenotype by antisense KAP expression.. Molecular and cellular biology.

[62] Sun Y, Wang L, Jiang M, Huang J, Liu Z (2010). Secreted phosphoprotein 1 upstream invasive network construction and analysis of lung adenocarcinoma compared with human normal adjacent tissues by integrative biocomputation.. Cell Biochem Biophys.

[63] Bae I, Rih JK, Kim HJ, Kang HJ, Haddad B (2005). BRCA1 regulates gene expression for orderly mitotic progression.. Cell Cycle.

[64] Stone R, Sabichi AL, Gill J, Lee IL, Adegboyega P (2010). Identification of genes correlated with early-stage bladder cancer progression.. Cancer Prev Res (Phila).

[65] Jones WM, Chao AT, Zavortink M, Saint R, Bejsovec A (2010). Cytokinesis proteins Tum and Pav have a nuclear role in Wnt regulation.. J Cell Sci.

[66] Mas VR, Maluf DG, Archer KJ, Yanek K, Williams B (2006). Differentially expressed genes between early and advanced hepatocellular carcinoma (HCC) as a potential tool for selecting liver transplant recipients.. Mol Med.

[67] Small M, Xu X, Zhou J, Zhang J, Sun J (2008). Scale-free networks which are highly assortative but not small world.. Phys Rev E Stat Nonlin Soft Matter Phys.

[68] Pau Ni IB, Zakaria Z, Muhammad R, Abdullah N, Ibrahim N (2010). Gene expression patterns distinguish breast carcinomas from normal breast tissues: the Malaysian context.. Pathol Res Pract.

[69] Ancona N, Maglietta R, Piepoli A, D'Addabbo A, Cotugno R (2006). On the statistical assessment of classifiers using DNA microarray data.. BMC Bioinformatics.

[70] Koziol JA, Feng AC, Jia Z, Wang Y, Goodison S (2009). The wisdom of the commons: ensemble tree classifiers for prostate cancer prognosis.. Bioinformatics.

[71] Parkinson H, Kapushesky M, Kolesnikov N, Rustici G, Shojatalab M (2009). ArrayExpress update–from an archive of functional genomics experiments to the atlas of gene expression.. Nucleic Acids Res.

[72] Irizarry RA, Hobbs B, Collin F, Beazer-Barclay YD, Antonellis KJ (2003). Exploration, normalization, and summaries of high density oligonucleotide array probe level data.. Biostatistics.

[73] Birney E, Andrews TD, Bevan P, Caccamo M, Chen Y (2004). An overview of Ensembl.. Genome Res.

[74] Carter SL, Brechbuhler CM, Griffin M, Bond AT (2004). Gene co-expression network topology provides a framework for molecular characterization of cellular state.. Bioinformatics.

[75] Newman ME (2006). Modularity and community structure in networks.. Proc Natl Acad Sci U S A.

[76] Drozdov I, Ouzounis CA, Shah AM, Tsoka S (2011). Functional Genomics Assistant (FUGA): a toolbox for the analysis of complex biological networks.. BMC research notes.

[77] Blondel VD, Guillaume J-L, Lambiotte R, Lefebvre E (2008). Fast unfolding of communities in large network.. J Stat Mech.

[78] Enright AJ, Van Dongen S, Ouzounis CA (2002). An efficient algorithm for large-scale detection of protein families.. Nucleic Acids Res.

[79] Ashburner M, Ball CA, Blake JA, Botstein D, Butler H (2000). Gene ontology: tool for the unification of biology. The Gene Ontology Consortium.. Nature genetics.

[80] Huang da W, Sherman BT, Lempicki RA (2009). Systematic and integrative analysis of large gene lists using DAVID bioinformatics resources.. Nat Protoc.

[81] Wex T, Treiber G, Lendeckel U, Malfertheiner P (2003). A two-step method for the extraction of high-quality RNA from endoscopic biopsies.. Clin Chem Lab Med.

[82] Boyault S, Rickman DS, de Reynies A, Balabaud C, Rebouissou S (2007). Transcriptome classification of HCC is related to gene alterations and to new therapeutic targets.. Hepatology.

