# Supplementary figures and images for: Functional and Topological Properties in Hepatocellular Carcinoma Transcriptome
Source: PLoS One. 2012 Apr 23;7(4):e35510. doi: 10.1371/journal.pone.0035510 (PMC3335123; doi:10.1371/journal.pone.0035510)

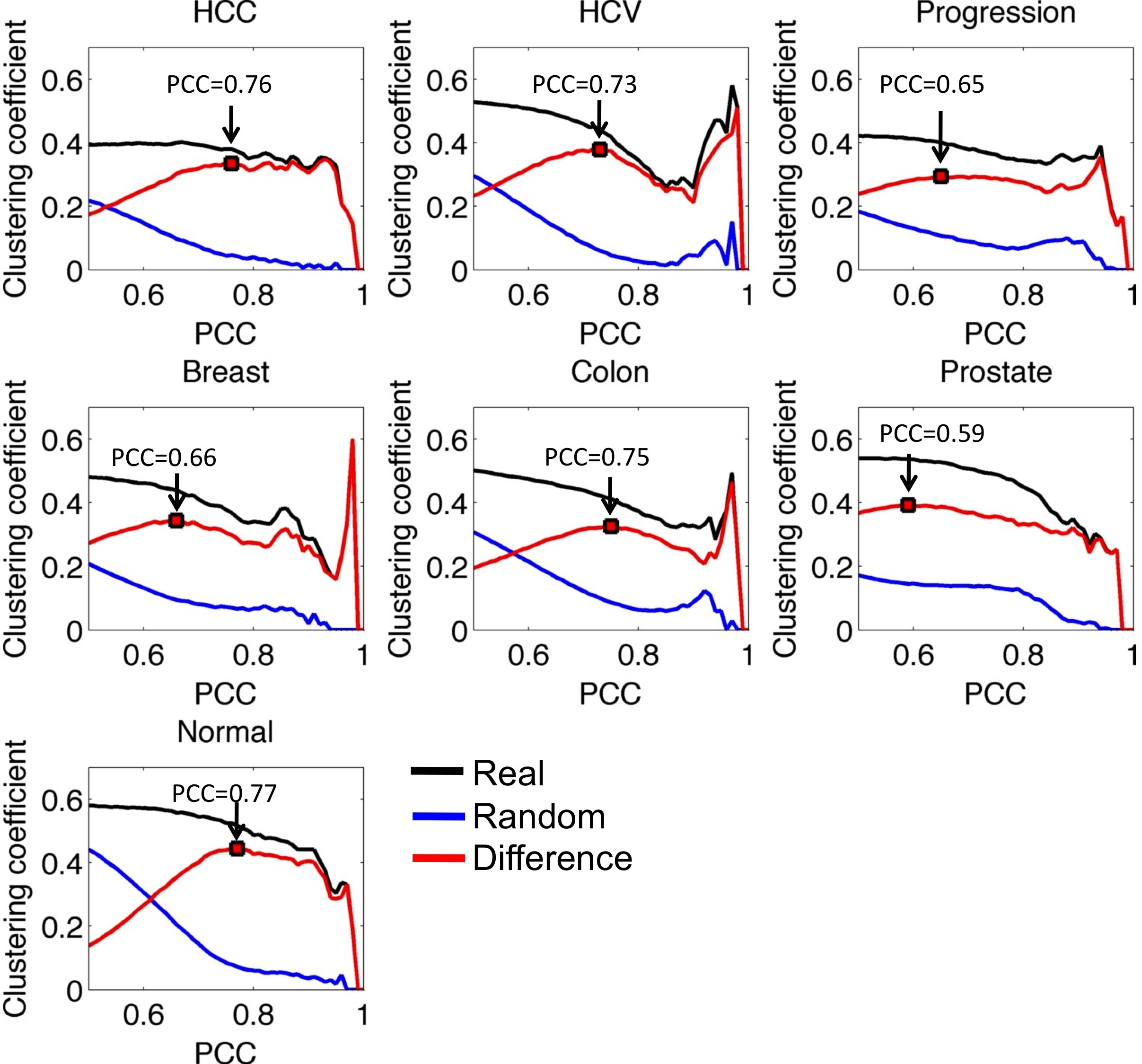

Supplement: Figure S1 — Selection of Pearson correlation coefficient threshold for gene co-expression network inference. Clustering coefficients of real (black) and random (blue) networks with identical node degree distributions were systematically measured for 0.50≤PCC≤1.0. Threshold was selected at the first local maximum of the difference (red) between the real and random clustering coefficients. (TIFF) [file pone.0035510.s001.tiff]

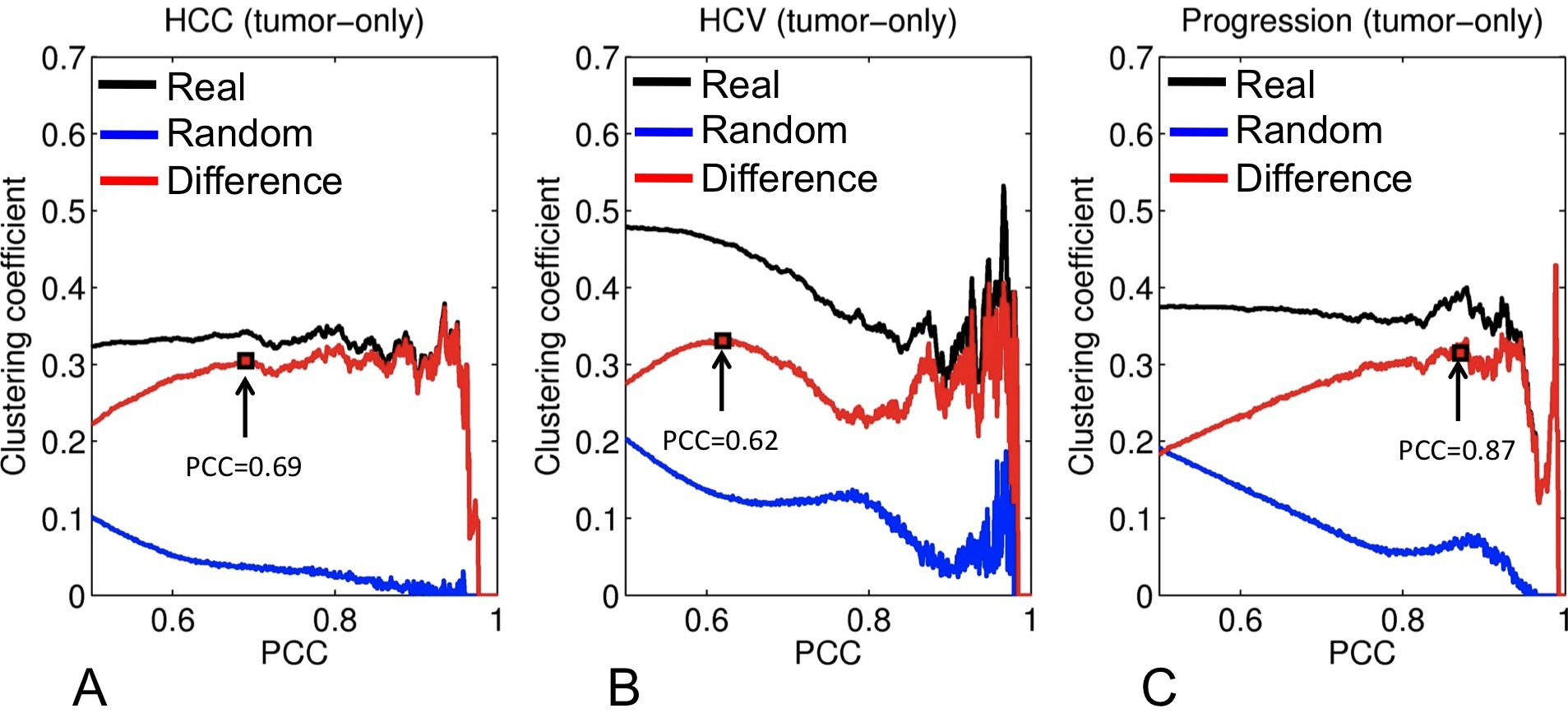

Supplement: Figure S2 — Selection of Pearson correlation coefficient threshold for gene co-expression networks of HCC microarrays without normal liver. Clustering coefficients of real (black) and random (blue) networks with identical node degree distributions were systematically measured for 0.50≤PCC≤1.0. Threshold was selected at the first local maximum of the difference (red) between the real and random clustering coefficients. (TIFF) [file pone.0035510.s002.tiff]
